# Scientometric analysis of the Puerto Rico Clinical and Translational Research Consortium (PRCTRC) research publications, 2010–2018

**DOI:** 10.1017/cts.2020.43

**Published:** 2020-05-08

**Authors:** Mariela Torres-Cintrón, Carlamarie Noboa-Ramos, Zulmarie De Pedro-Serbia, Mariela Lugo-Picó, Lorena González-Sepúlveda, Naydi Pérez-Ríos, Margarita Irizarry-Ramírez

**Affiliations:** Puerto Rico Clinical and Translational Research Consortium, University of Puerto Rico Medical Sciences Campus, San Juan, Puerto Rico

**Keywords:** Metrics, publications, collaborations, scientometrics, citations

## Abstract

We analyzed the publication productivity supported by the Puerto Rico Consortium for Clinical and Translational Research (PRCTRC) using the structured process of scientometrics. The objective of this study was to evaluate the impact of the research and collaborations as presented in publications. Manuscripts published from 2010 to 2018 and that had the PRCTRC award number and a PMCID number were retrieved from the Science Citation Index database. Scientometric indicators included h-index (HI), average citation (AC), collaboration coefficient (CC), collaboration index (CI), and degree of collaboration (DC) analysis, and relative citation ratio (RCR) was done with Web of Science Platform, iCite, and Stata software. Joinpoint Trend Analysis Software was used to calculate the annual percent change (APC). From 2010 to 2018, 341 publications were identified with an average of 38 publications per year and a total of 3569 citations excluding self-citations. A significant growth (APC: 17.76%, *P* < 0.05) of scientific production was observed. The overall HI was 31, and the AC per item was 11.04. The overall CC was 0.82, the CI was 8.59, and the DC was 99.1%. This study demonstrates a statistically significant increase in the PRCTRC scientific production. Results allow for the assessment of the progress resulting from the provided support and to plan further strategies accordingly.

## Introduction

The Puerto Rico Clinical and Translational Research Consortium (PRCTRC) was established on September 2010 with the idea of implementing productive collaborations among local institutions and to have a broader impact across all levels of research in health disparities in Puerto Rico. Supported through the National Center for Research Resources and the National Institute of Minority Health and Health Disparities (NIMHD) of the National Institutes of Health (NIH) (U54 RR026139, P20 RR011126, U54 MD007587), the PRCTRC is a research infrastructure and capacity building partnership among the University of Puerto Rico Medical Sciences Campus (UPR-MSC), a public academic health sciences center as the leader; the Ponce Health Sciences University (PHSU), and Universidad Central del Caribe (UCC) [1]. PRCTRC has fostered a synergistic environment to support research across the clinical and translational research continuum in four primary clinical focus areas: HIV/AIDS, neuroscience, cardiovascular and metabolic disease, and cancer.

Research publications represent the public cumulative record of science, documenting empirical results and providing a forum for theorizing, debate, and the gradual advance of scientific knowledge [2]. Although publications are not the end point of scientific research, it is impossible to imagine translating research into knowledge or practice without the manifestation of research in publications [2]. The quantitative analysis of publications is known as bibliometrics. Bibliometrics can be defined as “the application of quantitative analysis and statistics to publications such as journal articles and their accompanying citation counts” [3]. Bibliometrics (sometimes called scientometrics) turns the main tool of science, quantitative analysis, on itself. The complexity is in the analysis and use of the numbers, for the statistics obtained can be understood as indicators of achievement or lack thereof [3].

Scientometric analysis provides a structured process to understand the dynamics of the sciences [4]. It is an important method of health science informatics research, which can be used for quantitative analysis of the intellectual structures and connections of scientific articles in a specific field [5,6]. This tool has the potential to contribute to a better understanding of the quantity, impact, and types of research that the PRCTRC supports. Collaboration between researchers is of great importance in the development of subject areas and in the dissemination of research results. It is a powerful form of interaction that allows for effective communication as well as the sharing of competence and other resources [7]. Collaboration metrics can help identify where collaboration has and has not taken place [3]. Such analysis can also reveal dynamic and influential points of collaboration, as well as those that have been less influential [3].

The Clinical and Translational Science Awards (CTSA) program is one of the most important initiatives in translational medical funding [8]. To provide a quantitative evaluation of the CTSA program, Zhang and collaborators [6,8] performed studies based on science mapping, scientometric analysis, and information extraction techniques. One of these studies quantitatively analyzed the scientific articles funded by the CTSA program. The results of the study showed that the quantitative productivities of the CTSA program had a stable increase since 2008. Also, the emerging trends of the research funded by the CTSA program covered clinical and basic medical research fields [8]. The quantitative evaluation of the efficiency and performance of the CTSA program has a significant referential meaning for the decision making of global translational medical funding [8]. The second study attempted to show the scientific output and impact, identify the specific core field and institute, and understand the academic status and benefit of translational medicine to evaluate quantitatively the efficiency and performance of translational medicine [6]. The research showed that translational medicine had significant scientific output and impact, core field and institute, and academic status and benefit [6]. Llewellyn and collaborators also evaluated publication and citation patterns for articles supported by CTSA hub investment over the first decade of the CTSA program [9]. The authors collected bibliometric data from PubMed, Web of Science InCites, and NIH iCite for articles citing any CTSA hub grants published from hub inception through 2016. They compiled data on publication and citation rates, and indexes of relative citation impact aggregated by hub funding year cohort. The study showed that the CTSA program is yielding a robust and growing body of influential research findings with consistently high indices of relative citation impact [9]. Another study of CTSA used complementary bibliometric approaches to assess the scope, influence, and interdisciplinary collaboration of publications supported by single CTSA hubs and those supported by multiple hubs, characterizes the CTSA consortium’s contributions to clinical and translational science, identifies content areas of strength, and provides evidence for the success of multihub collaborations. The two articles by Zhang focused on the top 10 CTSA funded institutions and the top 20 agencies for translational research and Puerto Rico was not part of that group. None of the abovementioned studies include the research at Puerto Rican institutions.

In Puerto Rico, two prior studies assessed the scientific research productivity of Puerto Rican cancer researchers, so prior bibliometric studies have been conducted but limited to cancer [10,11]. One of the articles presents the characteristics and trends of cancer publications in Puerto Rico’s biomedical journals and their relationship with the island’s cancer mortality [10]. This first study limits its analysis to the papers published in the Puerto Rico Health Sciences Journal and the Boletín de la Asociación Médica de Puerto Rico from 1903 to 2005. Bibliometric indicators studied included the number of authors and references by article, first author’s institutional affiliation and country, document type, and language [10]. A study by Calo and collaborators characterizes the trends in cancer-related research publications by authors affiliated to Puerto Rican institutions in recent decades [11]. They used the Science Citation Index (SCI) database from 1982 to 2009. Search criteria were that the author’s affiliation field contained some institution located in Puerto Rico and that the manuscripts were related to cancer research (according to keywords from the National Cancer Institute’ cancer definition). The indexes measured in the analysis included number and type of manuscript, scientific collaboration, author’s affiliation, and journal visibility [11].

In the current study, we are incorporating other metrics to understand the big picture of publication productivity supported by the Puerto Rico Consortium for Clinical and Translational Research (PRCTRC) using Scientometric and collaboration indicators. Our aim is to present a detailed scientific analysis of the PRCTRC impact as a generator of important contributions addressing health questions in an underserved population.

Here we present a summary of the research impact of the PRCTRC from 2010 to 2018. Measuring the research impact was done by assessment of the researchers’ performance and their contribution to the creation of knowledge and technology, using citation analysis and objective, quantitative data [3].

This examination has the potential to contribute to a richer understanding of the quantity, impact, and types of research the PRCTRC supports, providing evidence of the accomplishments and capacities of the institution.

## Methods

Peer-reviewed publications published from 2010 to 2018 were retrieved from the SCI database (October 2019). Our search criteria included publications that (1) acknowledge the PRCTRC support (i.e., award numbers U54RR026139, P20RR011126, U54MD007587) and (2) have a PMID number. To try to minimize the situation about the misprint award numbers, we created an algorithm with a series of combination of the awards numbers and then we verified one by one the papers retrieved.

For this study, the *Scientometric indicators* included citation indicators [i.e., papers count, citation counts, h-index (HI), and average citation (AC) per item) and collaboration indicators (i.e., collaboration index (CI), degree of collaboration (DC), and collaboration coefficient (CC)]. We stand by the use of the HI, as a simple and useful way to characterize the scientific output of a researcher which has been extensively used by others to establish comparisons of scientists, scientific journals, research teams, and research institutions and countries [12].

The *citation indicators* were calculated using the Web of Science Platform and iCite [13] program, and the *collaboration indicators* were calculated using Stata software for statistical inquiry. Joinpoint Trend Analysis Software and Excel were used to make the trends analysis for citation indicators. We also describe the principal research areas of the PRCTRC publications.

The indicators definitions are listed here [3,7] (Table 1).

**Table 1.** Indicators definitions

	Indicator	Definition	Measure
Citation indicators	*Paper counts*	Number of items published each year	Productivity
	*Citation counts*	The number of citations each year	Total recognition/influence
	*Average citation (AC) per item*	Is computed by dividing the sum of citations to some set of papers for a defined time period by the number of papers (paper count)	Efficiency
	*Hirsch index (H-index)*	The h-index is based on a list of publications ranked in descending order by the times cited. The value of h is equal to the number of papers (*N*) in the list that have *N* or more citations	Productivity (number of papers) and impact (number of citations) in one number
	*Annual percent change (APC)*	One way to characterize trends in publication rates over time	Trend analysis
	*Relative Citation Ratio (RCR)*	It is calculated as the citations of a paper, normalized to the citations received by NIH-funded publications in the same area of research and year.	Influence
Collaboration Indicators	*Collaboration coefficient (CC)*	Reflect both the mean number of authors per paper as well as the proportion of multiauthored papers	Authorship pattern
	*Collaboration index (CI)*	Measure of mean number of authors	Authorship and collaboration
	*Degree of collaboration (DC)*	Measure of proportion of multiauthored papers	Authorship and collaboration

The Web of Science Platform include the following databases: CCC (Current Contents Connect), WOS (Web of Science), INSPEC (Institution of Engineering and Technology), ZOOREC (Zoological Record), SCIELO (Scientific Electronic Library Online), DIIDW (Derwent Innovation Index), MEDLINE (National Library of Medicine premier life sciences database), RSCI (Russian Science Citation Index), KJD (Korean Journal Database), DRCI (Data Citation Index), BCI  = Book Citation Index (Science). The Web of Science database is one of the most authoritative and multidisciplinary platforms in the world, covering nearly all the leading scholarly articles and high-quality citation data.

## Results

**Fig. 1.**
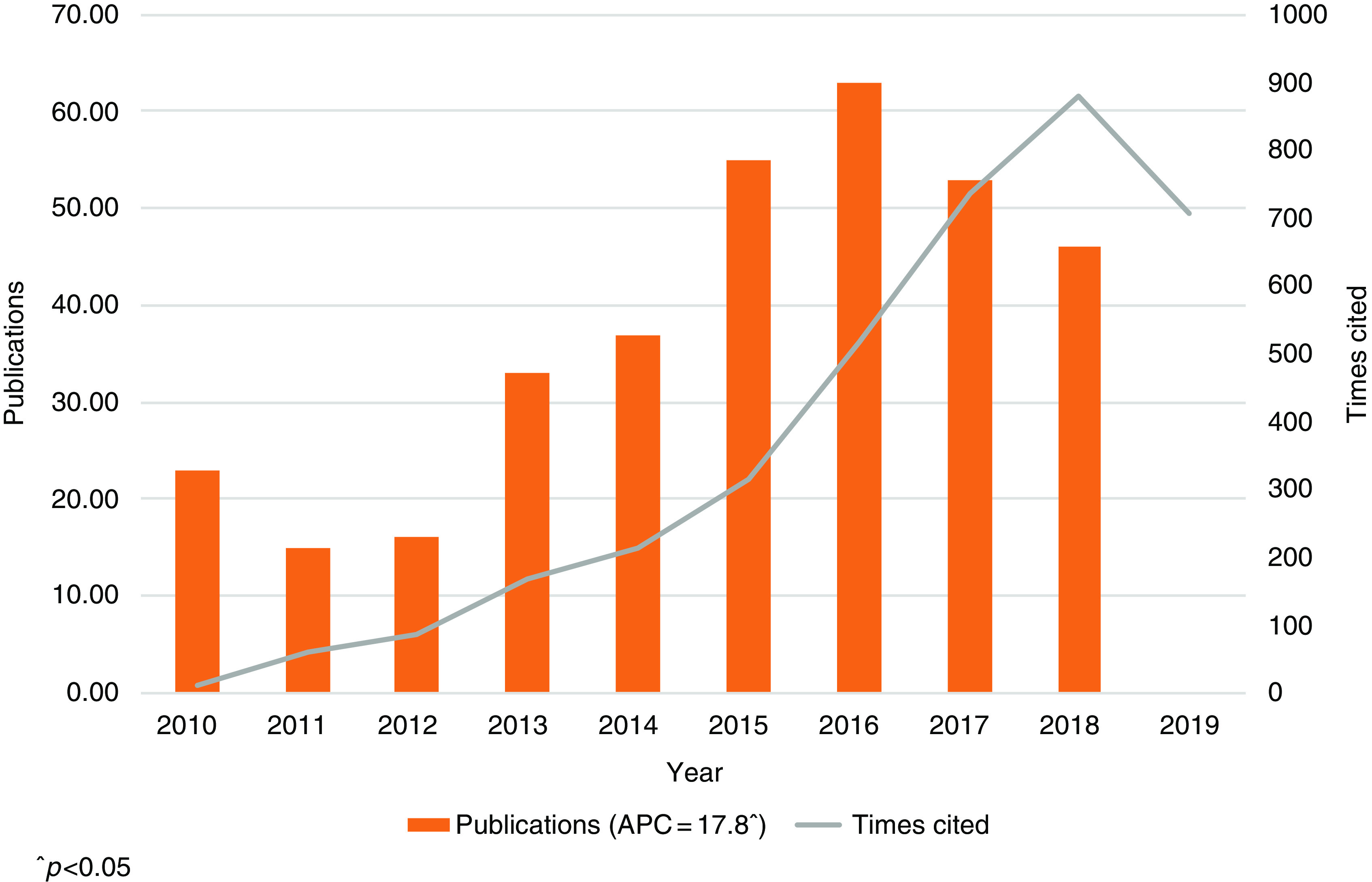
PRCTRC publication impact over time, 2010–2018.

A total of 341 peer reviewed PRCTRC supported publications were identified from 2010 to 2018 from all databases, with an average of 38 publications per year. During this period, a significant growth [annual percent change (APC): 17.76% *P* < 0.05] of scientific production was observed (Fig. 1). The overall HI was 31. The average number of citations per publication was 11.04. And the overall ACs per year was 376. The total number of times that all articles have been cited was 3763, but when self-citations were excluded it was 3569, for a difference of 194 publications. The relative citation ratio (RCR) showed a mean of 1.08 with a maximum of 9.77.

In terms of collaboration indicators, only three papers (0.87%) were single authored. Papers with four or more authors (87.1%) dominate the PRCTRC collaboration pattern. The average CC for the PRCTRC researchers during the studied time frame was 0.82. The highest CI with an average of 10.28 authors per paper was seen in year 2016 (Table 2).

**Table 2.** Quantification of publications by numbers of authors, collaboration coefficient (CC), and collaboration index (CI)

	Authorship pattern														
Year of publication	1	2	3	4	5	6	7	8	9	10 or more	No. of paper	CC	CI overall	CI (grouped after 4)	CI (grouped after 10)
2010	0	0	0	1	2	8	1	4	2	5	23	0.86	7.43	4.0	7.35
2011	0	2	1	2	1	3	3	1	0	2	15	0.78	6.07	3.67	5.80
2012	0	1	0	4	1	1	2	2	1	4	16	0.82	7.25	3.88	6.75
2013	1	1	6	1	5	6	2	2	2	7	33	0.79	7.24	3.67	6.18
2014	0	3	2	7	4	4	3	1	2	11	37	0.81	7.32	3.78	6.51
2015	0	4	2	7	8	4	6	3	4	17	55	0.83	8.51	3.82	6.87
2016	0	2	4	6	13	6	4	2	7	19	63	0.84	10.29	3.87	6.95
2017	2	1	5	4	7	3	5	3	4	19	53	0.81	9.34	3.75	7.04
2018	0	2	5	4	5	3	5	2	2	18	46	0.84	9.39	3.80	7.11
Total	3	16	25	36	46	38	31	20	24	102	341	0.82	8.59	3.81	6.82

The nine-year CI in PRCTRC papers was 8.59. And the CI (grouped after 4) was 3.81. The average DC between researchers in the investigated papers was 0.99 (Table 3).

**Table 3.** Authorship pattern with degree of collaboration measures (DC)

Authorship pattern	Number of publications	% of total publications	DC
Total number of single/multiauthored publication	341		
Number of co-authored publication (NM)	338		0.99
Number of single author publication (NS)	3	0.01	
Two authors publication	16	0.05	0.94
Three authors publication	25	0.07	0.96
Four authors publication	36	0.11	0.97
Five authors publication	46	0.13	0.98
Six authors publication	38	0.11	0.97
Seven authors publication	31	0.09	0.97
Eight authors publication	20	0.06	0.95
Nine authors publication	24	0.07	0.96
Ten and above authors publication	102	0.30	0.99

**Fig. 2.**
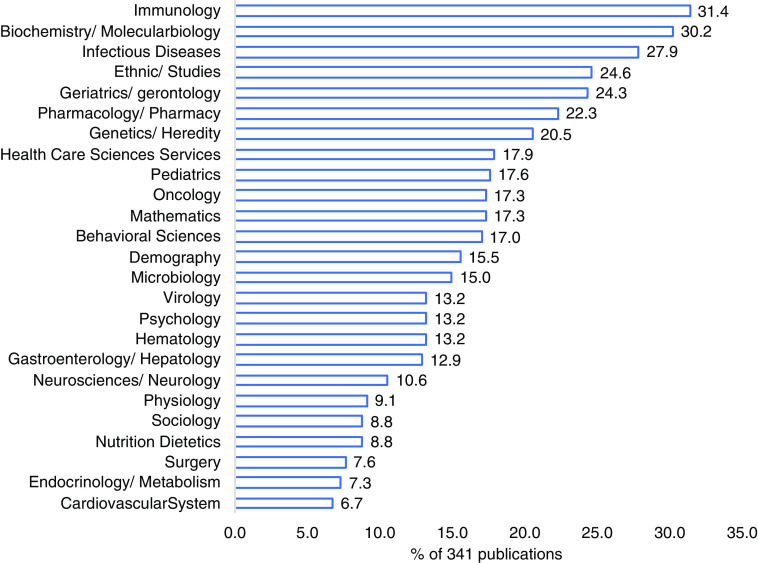
Top 25 PRCTRC research areas publications (341 publications).

The 12 principal research areas of the PRCTRC publications are: Immunology, Biochemistry/Molecular biology, Infectious Diseases, Ethnic/Studies, Geriatrics/gerontology, Pharmacology/Pharmacy, Genetics/Heredity, Health Care Sciences Services, Pediatrics, Mathematics, Oncology, Behavioral Sciences. Each of these research areas appears on at least 17% of the PRCTRC publications (Fig. 2).

## Discussion

In this study, scientometric analysis and collaboration measures were integrated into the evaluation of the efficiency and performance of the PRCTRC. In terms of Citation Indicators, the overall HI was 31, meaning that 31 papers in the list have 31 or more citations. This number reflects the productivity of authors based on their publication and citation records. The HI reflects not just the number of papers, or the number of citations; it also indicates the number of well-cited papers [14], and it is not influenced by a single highly cited paper because the HI can be determined for any population of articles. Very productive researchers in subject areas with high volume of publications and citations can show HI values over 100 at the peak of their scientific careers. Newer researchers in smaller subject areas can have HIs under 10 [14]. The HI is a particularly simple and useful way to characterize the scientific output of a researcher but also was applied to compare scientists, scientific journals, research teams, and research institutions and countries [12]. In terms of influence the RCR has a mean of 1.08 with a maximum of 9.77. The RCR indicates how a publication has been cited relative to other publications in its co-citation network, and this is assumed to be reflective of the article’s area of research. Our results showed an RCR of 1.0 which means that our papers on average have received the same number of citations as would be expected based on the NIH-norm [13].

According to this research, the overall quantitative productivities of the PRCTRC program had a stable increase since 2010. The PRCTRC publications underwent significant growth from 2010 to 2017 with the highest productivity peak observed during 2016. It is worth noting that during this period the PRCTRC incorporated a new service for Scientific Writing Editing. Professional Development Core’s Scientific Writing was involved in writing and editorial services for manuscripts and grants written by new/early investigators. In addition, the core provided short-term online courses and workshops to encourage and provoke new/early investigators to write and submit grant proposals and seminars targeted to clinical specialties to engage clinicians in research. We theorize that the support provided by this service could be an important factor to explain in part the fast increase during the years 2015–2016.

Unprecedented destruction from recent hurricanes in 2017, not seen since the 1930s, resulted in widespread damage and disruption of the infrastructure throughout the whole Puerto Rican territory, causing short-term and long-term impacts which will extend for many years [15,16]. The island faced the upsetting panorama of power failures and widespread interruption of cellphone and Internet service, which lasted, depending on the geographical location, weeks or months and in some areas almost a year (September 2017–August 2018). As we reviewed the publication’s data, we notice a marked reduction of published papers during 2017–2018 period, which could be explained by the abovementioned catastrophe.

Prior studies in Puerto Rico were in cancer [10,11]. In the first study, a total of 369 articles were retrieved during the period of 1903 to 2005. The primary results showed that the institutions with most publications were universities (39.6%), English was the predominant publication language (72.1%), and the principal document type was original papers (69.6%). Epidemiologic studies were the dominant study type (62.1%), and the most studied cancers were digestive (15.4%) and gynecologic (9.6%). Although the publication percent has increased since 1913 (APC = 1.2%), the mortality percent increased at a faster pace (APC = 2.7%). They concluded that although a growth in the number of cancer publications is observed in these journals, it does not parallel the increase in proportional mortality [10]. The second study showed that from 1982 to 2009, cancer-related papers authored by scientists located in Puerto Rico came to 451. Over the last three decades, the scientific production underwent significant growth (APC = 6.4%, *P*  < 0.05) with the highest peak between 2000 and 2009 (61.4% of all articles). Universities are the local institutional sector with the highest number of authors (81.4%), and the University of Puerto Rico is the most active center in this regard (68.5%). Forty-three percent of the manuscripts (*n* = 195) were published in 20 journals from which 14 are observed to have high visibility when compared to similar thematic journals [11].

The present study expands the knowledge of scientific impact and collaboration in Puerto Rico. As mentioned above, results highlight a strong scientific collaboration among PRCTRC affiliated investigators as shown by the high average CC of 0.82 and the high average DC of 0.991 that were found in our results. This could be explained by the multidisciplinary nature of the PRCTRC and of clinical and translational science [17–20]. The research areas of the PRCTRC publications evaluated in this study covered clinical and basic medical research, including the PRCTRC focus areas of HIV/AIDS, neuroscience, cardiovascular and metabolic disease, and cancer.

The main innovations of this study were to evaluate the efficiency and performance of the PRCTRC by using scientometric analytic methods, author collaborations, and to reveal the quantitative productivities and the research areas preferences. As shown, the application of the scientometric indicators will allow the PRCTRC to assess the research productivity, impact, and collaboration to design and implement interventions according to the program experience and needs. This study provided a better understanding of the quantity, impact, and types of research that the PRCTRC supports and provided evidence of the accomplishments and capacities of the institution. Results allow for the assessment of the progress resulting from the provided support and to plan further strategies accordingly.

The present article contributes to the literature when considering that other academic centers would like to gauge their output and progress against a comparison center. The paper also contributes in terms of filling a research gap into the change in publication output over time within a single center. However, there are limitations of the research. The purpose of this first paper is to present a description of the behavior of the scientific productivity using bibliometrics indicators. A limitation on the presented data and results is that it does not include independent variables that would be valuable in understanding why the selected outcome metrics change over time. In future analyses, we will include comparison analyses to try to explain the changes over time. Another limitation is that our study used traditional metrics for assessing the bibliometric impact. In future studies, we will use alternative metrics for assessing impact. Other limitation of this study is that it was very difficult to calculate the indicator at author level because the authors do not have a standardized way to be cited. For future studies, we must analyses each author and group the different ways of cites for the same author.

A complete understanding of citing, publishing, and collaboration patterns in Puerto Rico is critical to researchers, policy makers, and heath care professionals in order to make informed decisions about research priorities and to guide the Puerto Rico Clinical and Translational Research Consortium. Although our study provides important information on publication patterns in the publications that mention the number of the PRCTRC grant number and that are in Web of Science database, a complete analysis of PRCTRC publications published in other databases by researchers affiliated with PRCTRC must be done. To fully understand the total spectrum of clinical and translational research in Puerto Rico, it is important that future bibliometric studies also evaluate other scientific materials such as monographs, books, and thesis dissertations and presentation [11]. We also recommend that future bibliometric studies of clinical and translational research publications include citation analysis for authors affiliated to the PRCTRC. Finally, the scientometrics and collaboration characteristics of Puerto Rico should be compared to that of other Clinical and Translational Centers, to further understand our level of development in the area of Clinical and Translational Research. The evaluation of the scientometric measures will serve to other similar research consortia to determine areas of excellence, identify possible interventions to improve the landscape, and to define areas in which research has a room to grow.

## References

[1] Estape-Garrastazu ES , Noboa-Ramos C , De Jesus-Ojeda L , De Pedro-Serbia Z , Acosta-Perez E , Camacho-Feliciano DM. Clinical and translational research capacity building needs in minority medical and health science Hispanic institutions. Clinical and Translational Science 2014; 7: 406–412. PMC4213207. 24841800 10.1111/cts.12165 PMC4213207

[2] Schneider M , Kane CM , Rainwater J , et al. Feasibility of common bibliometrics in evaluating translational science. Journal of Clinical and Translational Science 2017; 1: 45–52. PMC5408837. 28480055 10.1017/cts.2016.8 PMC5408837

[3] Thomson Reuters. Whitepaper using bibliometrics: A guide to evaluating research performance with citation data. Thomson Reuters, 2008.

[4] Börner K , Glänzel W , Scharnhorst A , van den Besselaar P. Modeling science: studying the structure and dynamics of science. Scientometrics 2011; 89: 347–348.

[5] Chen C , Hu Z , Liu S , Tseng H. Emerging trends in regenerative medicine: a scientometric analysis in CiteSpace. Expert Opinion on Biological Therapy 2012; 12: 593–608. 22443895 10.1517/14712598.2012.674507

[6] Zhang Y , Diao T , Wang L. Quantitative evaluation of translational medicine based on scientometric analysis and information extraction. Clinical and Translational Science 2014; 7: 465–469. 25079489 10.1111/cts.12193 PMC5350927

[7] Sangam S , Arali U. Growth versus scientific collaboration in the field of genetics: a scientometrics analysis. Collnet Journal of Scientometrics and Information Management 2016; 10: 9–19.

[8] Zhang Y , Wang L , Diao T. The quantitative evaluation of the Clinical and Translational Science Awards (CTSA) program based on science mapping and scientometric analysis. Clinical and Translational Science 2013; 6: 452–457. 24330689 10.1111/cts.12078 PMC5350956

[9] Llewellyn N , Carter DR , Rollins L , Nehl EJ. Charting the publication and citation impact of the NIH Clinical and Translational Science Awards (CTSA) program from 2006 through 2016. Academic Medicine 2018; 93: 1162–1170. PMC6028299. 29298181 10.1097/ACM.0000000000002119 PMC6028299

[10] Ortiz AP , Calo WA , Suárez-Balseiro C , Maura-Sardo M , Suárez E. Bibliometric assessment of cancer research in Puerto Rico, 1903–2005. Revista Panamericana de Salud Pública 2009; 25: 353–361. PMC3031111. 19531324 10.1590/s1020-49892009000400010 PMC3031111

[11] Calo WA , Suárez-Balseiro C , Suárez E , Soto-Salgado M , Santiago-Rodríguez EJ , Ortiz AP. Assessing the scientific research productivity of Puerto Rican cancer researchers: bibliometric analysis from the Science Citation Index. Puerto Rico Health Sciences Journal 2010; 29: 250–255. PMC5798447. 20799512 PMC5798447

[12] Mester G. Rankings scientists, journals and countries using h-index. Interdisciplinary Description of Complex Systems 2016; 14: 1–9.

[13] Hutchins BI , Hoppe TA , Meseroll RA , Anderson JM , Santangelo GM. Additional support for RCR: a validated article-level measure of scientific influence. PLOS Biology 2017; 15: e2003552. 28968381 10.1371/journal.pbio.2003552 PMC5624567

[14] Web of Science: h-index information. Clarivate Analytics [Internet], 2019. (https://support.clarivate.com/ScientificandAcademicResearch/s/article/Web-of-Science-h-index-information?language=en_US)

[15] Alcorn T. Puerto Rico’s health system after Hurricane Maria. Lancet 2017; 390: e24. 29131798 10.1016/S0140-6736(17)32591-6

[16] Zorrilla CD. The view from Puerto Rico – hurricane Maria and its aftermath. The New England Journal of Medicine 2017; 377: 1801–1803. 29019710 10.1056/NEJMp1713196

[17] Ameredes BT , Hellmich MR , Cestone CM , et al. The Multidisciplinary Translational Team (MTT) model for training and development of translational research investigators. Clinical and Translational Science 2015; 8: 533–541. 26010046 10.1111/cts.12281 PMC4626313

[18] Estape ES , Quarshie A , Segarra B , et al. Promoting diversity in the clinical and translational research workforce. Journal of the National Medical Association 2018; 110: 598–605. PMC6230318. 30129489 10.1016/j.jnma.2018.03.010 PMC6230318

[19] Noboa-Ramos C. Integration of social network analysis into Puerto Rico Clinical and Translational Research Consortium (PRCTRC) evaluation plan. *31st Annu Int Conf Am Eval Assoc*, Washington, DC, 2017.

[20] Siamaki S , Geraei E , Zare-Farashbandi F. A study on scientific collaboration and co-authorship patterns in library and information science studies in Iran between 2005 and 2009. Journal of Education and Health Promotion 2014; 3: 99. 25250365 10.4103/2277-9531.139681 PMC4165092

